# Release of Type 2 Cytokines by Epithelial Cells of Nasal Polyps

**DOI:** 10.1155/2016/2643297

**Published:** 2016-12-29

**Authors:** Monica Boita, Caterina Bucca, Giuseppe Riva, Enrico Heffler, Giovanni Rolla

**Affiliations:** ^1^Department of Medical Sciences, University of Torino, Turin, Italy; ^2^1^st^ ENT Division, Department of Surgical Sciences, University of Torino, Turin, Italy; ^3^Respiratory Medicine and Allergology, Department of Clinical and Experimental Medicine, University of Catania, Catania, Italy

## Abstract

*Background*. T2 inflammation of chronic rhinosinusitis with nasal polyps (CRSwNP) may be influenced by epithelial cytokines release (TSLP, IL-25, and IL-33). We investigated the release of TSLP, IL-25, and IL-33 by epithelial CRSwNP cells compared to epithelial sinus mucosa cells of patients with chronic rhinosinusitis without nasal polyps (CRSsNP).* Methods*. IL-25, IL-33, and TSLP were measured by ELISA in the supernatant of cell cultures derived by CRSwNP (9 patients, 6 atopic) and CRSsNP (7 patients, 2 atopic) in baseline condition and following stimulation with* Dermatophagoides pteronyssinus *(DP),* Aspergillus fumigatus* (AF), and poly(I:C).* Results*. CRSwNP epithelial cells released increased levels of IL-25 (from 0.12 ± 0.06 pg/ml to 0.27 ± 0.1 pg/ml, *p* < 0.01) and TSLP (from 0.77 ± 0.5 pg/ml to 2.53 ± 1.17 pg/ml, *p* < 0.001) following poly(I:C) stimulation, while CRSsNP epithelial cells released increased levels of IL-25 and IL-33 following AF and DP stimulation, respectively (IL-25: from 0.18 ± 0.07 pg/ml to 0.51 ± 0.1 pg/ml, *p* < 0.001; IL-33: from 2.57 ± 1.3 pg/ml to 5.7 ± 3.1 pg/ml, *p* < 0.001).* Conclusions*. CRSwNP epithelial cells release TSLP and IL-25 when stimulated by poly(I:C) but not by DP or AF, suggesting that viral infection may contribute to maintain and amplify the T2 immune response seen in CRSwNP.

## 1. Introduction

Chronic rhinosinusitis without nasal polyps (CRSsNP) and chronic rhinosinusitis with nasal polyps (CRSwNP) have been considered as separate disease entities based on inflammatory and remodeling profiles with a predominance of TH1 cells in patients with CRSsNP and TH2 cells and eosinophils in patients with CRSwNP [1]. Recent studies have questioned this dichotomization of CRS [2], by reporting a wider spectrum of immunologic profiles. CRSwNP is generally characterized by type 2 inflammation with pronounced eosinophilia and the presence of high levels of IL-5 and IL-13 in Western countries, while neutrophilic and proinflammatory cytokines have been reported to be predominant in Chinese patients with nasal polyps [3], specifically in those patients who were IL-5/IL-17/IFN-gamma negative [4]. On the other hand, CRSsNP is no longer considered to be sustained by type 1 inflammation only, as initially reported by Van Zele et al. [5]. Heterogeneous inflammation may be observed in CRSsNP, with different predominant cytokines, such as not only IFN-gamma and IL-17A but also IL-5, similar to CRSwNP [2].

Epithelial cells are no more considered a mere physical barrier in the mucosal sites, but they play also a key role in the initiation and regulation of innate and adaptive immune responses. Thymic stromal lymphopoietin (TSLP) and other epithelial derived cytokines (interleukin-25, IL-25 and interleukin-33, IL-33), the so-called tissue cytokines, constitute a complex network for the regulation of immunity and inflammation. The expression of such cytokines by epithelial cells may be induced by different exogenous/endogenous stimuli, as well as pathogens, traumas, infections, allergens, Toll-like receptor ligands (TLR ligands), proinflammatory, and T2 cytokines [6, 7].

The response of nasal epithelial cells to diverse stimuli such as inhaled allergens and nonallergic triggers may play a role in the pathogenesis of chronic inflammatory diseases, such as chronic rhinosinusitis without nasal polyps (CRSsNP) and chronic rhinosinusitis with nasal polyps (CRSwNP). TSLP has been found highly expressed in sinus mucosa of patients with CRSwNP [8–11] and epithelial production of IL-25, IL-33, and TSLP has emerged as critical epithelial factors that can initiate and amplify airway inflammation [12, 13].

Viral infection may stimulate epithelial cells to produce type 2 cytokines driving a biased inflammation toward a type 2 immune response. The role of virus infection in the pathogenesis of nasal polyps is not clear, but it has been shown that respiratory virus genomes are commonly found in in secretions and tissue samples from patients with CRS [14]. To test the hypothesis that viral infection would induce IL-25, IL-33, and TSLP release in epithelia derived from CRSwNP patients, but not CRSsNP patients, we measured the production of TSLP, IL-25, and IL-33 by epithelial cells derived from nasal polyps of CRSwNP, in vitro exposed to poly(I:C), a synthetic analog of viral dsRNAi, compared to epithelial cells derived from sinus mucosa of CRSsNP.

Sinus bioptic samples were also stimulated with common allergens, like* Aspergillus* and house dust mites, known to activate epithelial cells due to their protease activity [15, 16].

## 2. Materials and Methods

### 2.1. Patients

Sixteen consecutive Caucasian patients, 8 male, mean age of 49.33 ± 15.86 years (range 22–80 years) recruited for functional endoscopic sinus surgery (FESS), at the 1st ENT Division of the University of Turin, Italy, were enrolled in this study, approved by the local ethics committee, having obtained the written informed consent by all of them. All the patients were affected by CRSwNP (*n* = 9, 6 atopic) or CRSsNP (*n* = 7, 2 atopic). Diagnosis of CRSwNP and CRSsNP was based on symptoms and fiber optical examination and by sinus CT scan, according to European Position Paper on Rhinosinusitis 2012 criteria [1].

Skin prick tests for a panel of 14 inhalant standard allergens (ALK Abello', Hørsholm, Denmark) were performed in all included patients. Atopy was defined as a wheal diameter 3 mm or greater in the presence of expected results in control solutions (histamine dihydrochloride 20 mg/ml as a positive and solvent as a negative control). Measurement of specific IgE antibodies against common allergens (CAP-Phadia, Uppsala, Sweden) was performed in patients in whom skin prick tests could not be performed.

None of our subjects had current respiratory tract infection and none of them was treated with systemic or nasal corticosteroids or other medications in the four weeks prior to inclusion.

The study was approved by our Institutional Ethics Committee and all patients signed an informed consent to use their clinical data for scientific purpose.

### 2.2. Sinus Biopsies

Biopsy specimens were collected from nasal polyps of CRSwNP and in vitro exposed to different stimuli, compared to epithelial cells derived from ethmoid tissue of CRSsNP. All specimens were immediately placed in BEGM medium (Bronchial Epithelial Cell Growth Media, Clonetics-Lonza) with no serum but supplemented with penicillin 100 U/ml, streptomycin 100 *µ*g/ml, and glutamine.

### 2.3. Epithelial Cell Culture

Tissue specimens were obtained during surgery, from nasal polyps and ethmoid tissue of patients with CRSwNP and CRSsNP, respectively. The samples were immediately placed in BEGM medium (Bronchial Epithelial Cell Growth Media, Clonetics-Lonza) with no serum, supplemented with penicillin 100 U/ml, streptomycin 100 *µ*g/ml, and glutamine. Afterwards the samples were washed twice in NaCl solution and cut into small pieces (~1 mm^2^). Diced specimens were then plated (density, 9 pieces/6-well tissue culture dish) in BEGM medium and incubated in a humidified 5% CO_2_ atmosphere at 37°C, until a monolayer of epithelial-like cells was observed to be confluent, usually after 13 ± 2 days. Then the explanted tissues were removed, and cells were trypsinized and replated into 6-well tissue culture dish at a final volume of 1.5 ml of fresh BEGM medium. The medium was changed every 3 days for 2-3 weeks until 90% confluence was obtained, usually after 17 ± 3 days, when the cells were stimulated overnight. To assess purity of the culture, cells were trypsinized and washed and one aliquot was analyzed for EpCAM (epithelial cells marker) expression with flow cytometry technique and another aliquot was examined in the Bürker chamber for counting the number of cells.

### 2.4. Epithelial Cell Stimulation and EpCAM Evaluation

Before stimulation, media were removed and replaced with fresh media. Cells were stimulated with 25 *µ*g/ml poly(I:C), 1.6 *µ*g/ml of* Dermatophagoides pteronyssinus*, and 2.6 *µ*g/ml of* Aspergillus fumigatus* separately, for 24 h at 37°C in 5% CO_2_. As negative control epithelial cells were incubated with culture medium only. Stimulated cell cultures were centrifuged at 4°C, and the supernatants were collected and stored at −20°C until being analyzed. Subsequently, cells were trypsinized and washed and an aliquot was analyzed for EpCAM (epithelial cells marker) expression with flow cytometry technique; another aliquot was examined in the Bürker chamber for counting the number of stimulated cells. The median cells number/well was 49312 ± 14645, and the cells were always more than 90% EpCAM positive (94 ± 4 percentage of EpCAM positive cells). Staining for EpCAM was performed using EpCAM-PE antibody (BioLegend, San Diego, CA) on a Beckman Coulter XL cytometer.

### 2.5. Cytokine Production

Cell culture supernatants were analyzed for cytokine release by a Multiplex Bio-Plex Technology by Bioclarma srl (Turin, Italy). Samples were unfrozen overnight, then centrifuged, and analyzed for TSLP, IL-25, and IL-33. The assay was performed according to the manufacturer's instructions.

### 2.6. Statistics

Statistical analysis was performed by using GraphPad Prism 4.0c, GraphPad Software, Inc., CA, USA. Data are given as means ± SD. Differences between groups were tested by ANOVA followed by a post hoc test and an unpaired two-tailed Student's *t*-test and considered to be significant when *p* < 0.05. Differences in cytokine levels before and after stimulation were tested by paired *t*-test.

## 3. Results

**Figure 1:**
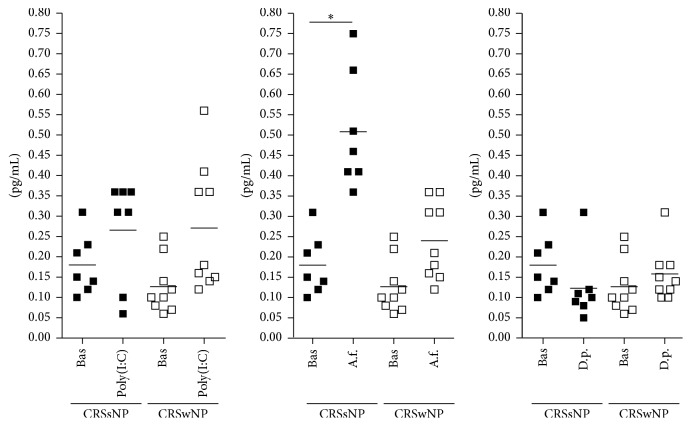
Mean and single values of IL-25 release in CRSsNP and CRSwNP patients. Bas = Baseline; A.f. =* A*.* fumigatus*; D.p. =* D*.* pteronyssinus*. ^*∗*^*p* < 0.001.

Clinical and demographic characteristics of the patients are reported in Table 1. Baseline production of epithelial cytokines was not different between epithelial cells derived by nasal polyps and epithelial cells derived by ostiomeatal mucosa of patients with CRSsNP (TSLP: 0.76 ± 0.48 pg/ml versus 0.61 ± 0.48 pg/ml; IL-25: 0.13 ± 0.07 pg/ml versus 0.18 ± 0.07 pg/ml; IL-33: 2.25 ± 1.26 pg/ml versus 2.56 ± 1.27 pg/ml, resp.). All the results are shown in Figure 1 (IL-25), 2 (TSLP), and 3 (IL-33).

### 3.1. Poly(I:C) Stimulation

**Figure 2:**
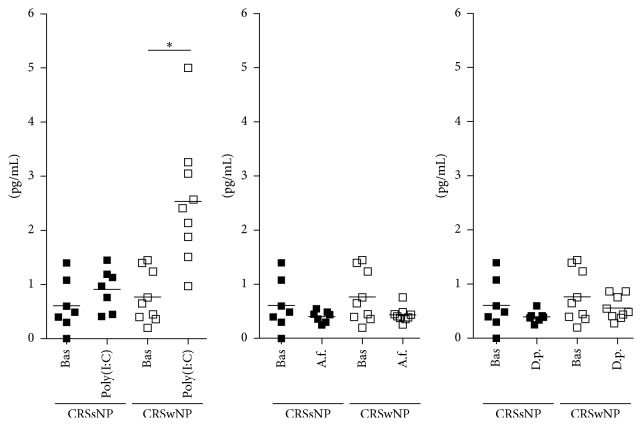
Mean and single values of TSLP release in CRSsNP and CRSwNP patients. Bas = Baseline; A.f. =* A*.* fumigatus*; D.p. =* D*.* pteronyssinus*. ^*∗*^*p* < 0.001.

**Figure 3:**
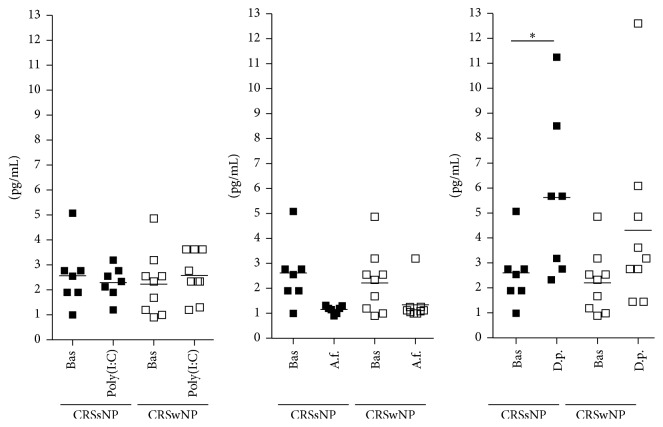
Mean and single values of IL-33 release in CRSsNP and CRSwNP patients. Bas = Baseline; A.f. =* A*.* fumigatus*; D.p. =* D*.* pteronyssinus*. ^*∗*^*p* < 0.001.

Following poly(I:C) stimulation, epithelial cells derived by nasal polyps released significantly higher amounts of IL-25 (from 0.12 ± 0.06 pg/ml to 0.27 ± 0.1 pg/ml, *p* < 0.01; Figure 1) and TSLP (from 0.77 ± 0.5 pg/ml to 2.53 ± 1.17 pg/ml, *p* < 0.001; Figure 2), with no change in IL-33 (from 2.25 ± 1.2 pg/ml to 2.57 ± 0.9 pg/ml, Figure 3). No change in cytokines release was observed in cultures of epithelial cells derived from ostiomeatal mucosa of patients with CRSsNP (IL-25: from 0.18 ± 0.07 pg/ml to 0.26 ± 0.1 pg/ml; TSLP: from 0.61 ± 0.5 pg/ml to 0.91 ± 0.4 pg/ml; IL-33: from 2.56 ± 1.3 pg/ml to 2.3 ± 0.6 pg/ml).

### 3.2. Allergen Stimulation

Following* A. fumigatus* and* D. pteronyssinus* stimulation, epithelial cells derived from ostiomeatal mucosa of patients with CRSsNP released significantly higher amounts of IL-25 and IL-33, respectively, with no change in TSLP (*A. fumigatus* stimulation, IL-25: from 0.18 ± 0.07 pg/ml to 0.51 ± 0.1 pg/ml, *p* < 0.001;* D. pteronyssinus* stimulation, IL-33: from 2.56 ± 1.3 pg/ml to 5.7 ± 3.1 pg/ml, *p* < 0.001;* A. fumigatus* and* D. pteronyssinus* stimulation, TSLP: from 0.61 ± 0.5 pg/ml to 0.4 ± 0.1 pg/ml and from 0.61 ± 0.5 pg/ml to 0.4 ± 0.1 pg/ml, resp.; see Figures 1, 2, and 3). Following* A. fumigatus* and* D. pteronyssinus* stimulation, no change in cytokines release was observed in epithelial cells derived by nasal polyps of patients with CRSwNP (*A. fumigatus* stimulation: IL-25 from 0.12 ± 0.06 pg/ml to 0.24 ± 0.09 pg/ml; IL-33 from 2.25 ± 1.2 pg/ml to 1.34 ± 0.7 pg/ml; TSLP from 0.77 ± 0.5 pg/ml to 0.43 ± 0.1 pg/ml;* D. pteronyssinus* stimulation: IL-25 from 0.12 ± 0.06 pg/ml to 0.15 ± 0.06 pg/ml; IL-33 from 2.25 ± 1.2 pg/ml to 4.31 ± 3.4 pg/ml; TSLP from 0.77 ± 0.5 pg/ml to 0.56 ± 0.2 pg/ml; see Figures 1, 2, and 3).

## 4. Discussion

We report that epithelial cells derived from nasal polyps of patients with CRSwNP release TSLP and IL-25 when specifically stimulated by poly(I:C), while they could not be activated by the stimulation with* Dermatophagoides pteronyssinus* or* Aspergillus fumigatus* extracts.

Poly(I:C) is a synthetic analog of viral dsRNA [17], which have been shown to closely mimic inflammatory responses associated with viral infection of airway epithelial cells [18]. Experimental data showed that TSLP may be induced by rhinovirus infection or by exposure to double stranded (ds) RNA (viral surrogate) in the lungs of allergic mice [19] and in human bronchial epithelial cells (HBEC) [20]. More recently Perez et al. reported increased levels of TSLP in nasal washes obtained from newborns, infants, and toddlers with PCR-confirmed acute rhinovirus infection, compared to controls with no infection [21].

TSLP and TSLP receptor have been found highly expressed in sinus mucosa of patients with CRSwNP [8–11], and a recent work by Golebski et al. [22] was able to demonstrate that the TLR3 agonist and viral analog poly(I:C) were able to induce higher TSLP mRNA and protein expression in the epithelium isolated from nasal polyposis patients compared to epithelium form healthy controls, while a few and contrasting observations have been reported on the presence of IL-25 in CRS. IL-25 mRNA has been reported to be significantly elevated in ethmoid sinuses of CRSwNP compared to controls and CRSsNP by Lam et al. [23]. These authors found that the increased expression of IL-25 they observed in nasal polyp tissues of patients with CRSwNP correlated with worse computerized tomography scores and blood eosinophilia. On the other hand, Miljkovic et al. reported that IL-25 mRNA was significantly decreased in nasal polyps compared to ethmoid sinuses of controls [24]. Further study will be required to investigate the role of IL-25 in CRSwNP.

Our observation suggests that viral infections may enhance the T2 biased immunologic response of nasal polyps through the release of TSLP and IL-25 by epithelial cells. The function of these cytokines includes induction of a biased T2 innate response, through the engagement of specific receptors expressed on mast cells and basophils, as well innate type lymphocytes (ILC2). The effect of viruses on the interaction of epithelial cells and mast cells has been explored in vitro. Nagarkar et al. found that airway epithelial cells directly promoted production of type 2 cytokines in mast cells during viral infection through the production of TSLP. The same authors could not detect IL-33 protein in poly(I:C) stimulated or influenza infected epithelial cells, in agreement with our results [25].

The role of viral infection in CRSwNP is not clear, but the frequency of respiratory virus detection in secretions and tissue samples from patients with CRS is up to 40%, when real-time polymerase chain reaction is used, according to a recent report [14]. Either or not viral infections are pathogenetically involved in polyps development, our results suggest that the interaction between virus and epithelial cells of nasal polyps may enhance the T2 immune response driving the eosinophilic inflammatory response characteristically observed in CRSwNP.

The epithelial cells derived from sinus tissue of patients with CRSsNP, but not those derived by polyps, responded to* Aspergillus fumigatus *and* Dermatophagoides pteronyssinus *exposure with the production of IL-25 and IL-33 cytokines, but not TSLP, with no difference between atopic and nonatopic subjects. Both* Aspergillus fumigatus* and* Dermatophagoides pteronyssinus *contain proteolytic allergens that cause oxidative stress and epithelial damage independently on sensitization. Proteases have been shown to initiate the generation of reactive oxygen species (ROS) by epithelial cells, leading to oxidation of lipids, signaling through TLR4 and producing T2 cytokines [26].

Surprisingly we could not observe any effect of allergen stimulation on the release of epithelial cytokines from epithelial cells derived from nasal polyps.

A possible explanation for the different response of epithelial cells derived from nasal polyps and sinus tissue of CRSsNP tissue when exposed to poly(I:C) and allergens may be found in the reported different expression of TLRs. Wang et al. [27] reported that nasal polyp epithelial cells mainly express TLR3, which is engaged by poly(I:C) [28], while expression of TLR4 was very low. On the other hand, mRNA expression of TLR4 has been reported to be significantly increased in chronic rhinosinusitis tissues compared with that in nasal polyps [29] and TLR4 was overexpressed in the epithelium of chronic rhinosinusitis, evaluated by confocal analysis [30]. CRSsNP is a heterogeneous disease and the frequency of type 2 inflammation has been reported to be even higher than type 1 inflammation in Western world. So it is not surprising that the epithelial cells derived from sinus cells of patients with CRSsNP were found to release IL-25 an IL-33, which are considered TH2 cytokines, when the cells were exposed to proteases contained in* A. fumigatus* and* D. pteronyssinus* extracts. Similar results have been obtained by Kouzaki et al., who observed IL-33 release from bronchial epithelial cells exposed to Alternaria [31].

Actually the biological effects of IL-25 and IL-33 depend on the tissue environment. For example, ILC2s activated by IL-25 and IL-33 showed reduced Th2 cytokine secretion in the presence of plate-bound E-cadherin [32], suggesting that cell-cell adhesion may play a regulatory role in the biological effects of these cytokines. In cystic fibrosis IL-33 has been shown to synergize with chemoattractants to promote neutrophil recruitment that might be more harmful than beneficial, like in CRSsNP [33]. IL-33 biology has evolved and expanded from its predominant role in allergic pathology to tissue repair and fibrosis [34].

In conclusion, our data suggest that viral infection may contribute to maintaining and amplifying the T2 immune response commonly seen in CRSwNP, through the release of TSLP and IL-25 by epithelial cells of polyps. The role of IL-25 and IL-33 released by sinus epithelial cells exposed to proteases stimulation needs to be further investigated.

## Figures and Tables

**Table 1:** Demographic and clinical characteristics of patients.

Patient number	Age (years)	Gender	Atopy	Asthma	Sensibilization	Clinical diagnosis	Surgery
1	22	Male	Yes	No	Grass and olive tree pollen; house dust mite; cat and dog epithelia	CRSwNP	Polypectomy, middle antrostomy, ethmoidectomy
2	53	Female	No	No	/	CRSwNP	Polypectomy, middle antrostomy, ethmoidectomy
3	36	Male	No	No	/	CRSwNP	Polypectomy, middle antrostomy, ethmoidectomy
4	62	Female	Yes	No	House dust mite; cat epithelia	CRSwNP	Polypectomy, middle antrostomy, ethmoidectomy
5	38	Male	Yes	Yes	Grass pollen	CRSwNP	Polypectomy, middle antrostomy, ethmoidectomy
6	55	Female	Yes	No	Cat epithelia; grass pollen; moulds	CRSwNP	Polypectomy, middle antrostomy, ethmoidectomy
7	60	Male	Yes	No	Grass and weed pollen; house dust mite	CRSwNP	Polypectomy, middle antrostomy, ethmoidectomy
8	47	Female	Yes	Yes	Grass, olive, hazelnut, and plane tree pollen; house dust mite	CRSwNP	Polypectomy, middle antrostomy, ethmoidectomy
9	59	Female	No	No	/	CRSwNP	Polypectomy, middle antrostomy, ethmoidectomy
10	56	Male	Yes	Yes	House dust mite; cat epithelia	CRSsNP	Middle antrostomy, ethmoidectomy
11	40	Female	Yes	No	House dust mite; cat and dog epithelia	CRSsNP	Middle antrostomy, ethmoidectomy
12	42	Female	No	No	/	CRSsNP	Middle antrostomy, ethmoidectomy
13	27	Male	No	No	/	CRSsNP	Middle antrostomy, ethmoidectomy
14	46	Female	No	No	/	CRSsNP	Middle antrostomy, ethmoidectomy
15	70	Male	No	No	/	CRSsNP	Middle antrostomy, ethmoidectomy
16	80	Male	No	No	/	CRSsNP	Middle antrostomy, ethmoidectomy
